# RETRACTION: Proximal Femoral Nail Anti‐Rotation vs. Dynamic Hip Screws Decrease the Incidence of Surgical Site Infections in Patients With Intertrochanteric Fractures: A Meta‐Analysis

**DOI:** 10.1111/iwj.70302

**Published:** 2025-03-06

## Abstract

**RETRACTION:** DaiP. , ZhouH. , MaoX. , LiuC. , WangZ. , and KangY. , “Proximal Femoral Nail Anti‐Rotation vs Dynamic Hip Screws Decrease the Incidence of Surgical Site Infections in Patients with Intertrochanteric Fractures: A Meta‐Analysis,” International Wound Journal 20, no. 8 (2022): 3212–3220, 10.1111/iwj.14200.PMC1050226037095692

The above article, published online on 24 April 2023, in Wiley Online Library (http://onlinelibrary.wiley.com/), has been retracted by agreement between the journal Editor in Chief, Professor Keith Harding; and John Wiley & Sons Ltd. Following an investigation by the publisher, all parties have concluded that this article was accepted solely on the basis of a compromised peer review process. The editors have therefore decided to retract the article. The authors did not respond to our notice regarding the retraction.

**RETRACTION:** P. Dai , H. Zhou , X. Mao , C. Liu , Z. Wang , and Y. Kang , “Proximal Femoral Nail Anti‐Rotation vs Dynamic Hip Screws Decrease the Incidence of Surgical Site Infections in Patients with Intertrochanteric Fractures: A Meta‐Analysis,” International Wound Journal 20, no. 8 (2022): 3212–3220, 10.1111/iwj.14200.PMC1050226037095692

The above article, published online on 24 April 2023, in Wiley Online Library (http://onlinelibrary.wiley.com/), has been retracted by agreement between the journal Editor in Chief, Professor Keith Harding; and John Wiley & Sons Ltd. Following an investigation by the publisher, all parties have concluded that this article was accepted solely on the basis of a compromised peer review process. The editors have therefore decided to retract the article. The authors did not respond to our notice regarding the retraction.

